# Model-based trends in the estimated number of children affected by maternal cancer diagnosis or death in Finland in 1968–2022

**DOI:** 10.2340/1651-226X.2025.44072

**Published:** 2025-09-23

**Authors:** Kirsi M. Talala, Eetu E. Mäkinen, Karri J.M. Seppä, Tea M. Lallukka, Janne M. Pitkäniemi

**Affiliations:** a Cancer Society of Finland, Finnish Cancer Registry, Helsinki, Finland; b Faculty of Social Sciences, Tampere University, Tampere, Finland; c Department of Public Health, Faculty of Medicine, University of Helsinki, Helsinki, Finland

**Keywords:** Child, orphans, mothers, neoplasms, trend, estimation, registries

## Abstract

**Background and purpose:**

Cancer is among the leading causes of premature death worldwide, and, in Finland, it is the most common cause of death among women aged 15–64 years who may be parenting minor children. We aim to estimate how many children are affected by maternal cancer or cancer death and if this has changed in Finland.

**Patient/material and methods:**

We used female cancers (Finnish Cancer Registry), cancer deaths, fertility rates in women and mortality rates in children (Statistics Finland) to calculate the model-based annual trend estimates of new and prevalent children under 18 years whose mother was diagnosed with cancer and new and prevalent orphans by maternal cancer type in Finland between 1968 and 2022.

**Results:**

The estimated rate of children whose mother was diagnosed with cancer increased 1.3% annually since 1996. In 2022, the rates of new and prevalent children with maternal cancer were 218.4 and 1522.4 per 100,000, corresponding to 2,334 and 16,803 children. On the contrary, the estimated rate of new orphans due to maternal cancer mortality decreased 1.2% annually since 1998. In 2022, the age-standardised rates of new and prevalent orphans were 26.4 and 166.7 per 100,000 children, corresponding to 285 and 1,850 orphans due to maternal cancer mortality.

**Interpretation:**

We estimated that the rate of new orphans due to maternal cancer mortality has declined over the past decades, which has benefited children. However, the increase in cancer incidence among mothers with minor children showed an opposite trend, indicating more intergenerational consequences due to cancer.

## Introduction

Cancer continues to be a major public health issue [1] and among the leading causes of premature death (occurring at the ages of 30–70 years) worldwide [2, 3]. In Finland, cancer is the most common cause of death among women aged 15–64 years [4], accounting for 978 (crude death rate 58.3/100,000) deaths for the year 2022. Consequently, maternal cancer and cancer death may be experienced by children before they reach adulthood (18 years of age). Studies have shown that parental illness and death have long-term impacts on children’s well-being, also in high Human Development Index (HDI) countries, including Northern Europe [5–7].

A study conducted by Guida et al. [8] estimated globally the number of children under 18 years who became maternal orphans due to cancer. The number of orphan children due to cancer is the lowest in the higher HDI countries. It was estimated that 60,000 children in Europe became orphans due to their mother dying from cancer in 2020, corresponding to 7 new orphans per 100 cancer deaths in women or 38 orphans per 100,000 children. The largest numbers of maternal orphans in Europe were due to deaths from breast, respiratory (mainly lung) and cervical cancers.

In Finland, the age-standardised incidence of breast cancer increased 2.2% annually between 1990 and 1999. Since then, during the 2000s, this increase has begun to stabilise being 1.2% annually. Breast cancer mortality slowly increased until the early 1990s but has since declined [9]. Among female genital organ cancers, cervical cancer incidence has decreased by 80% from 1963 to 2022 [10]. However, the incidence of cervical cancer has increased since the 1990s among women aged 30–40 years. The three most common new cancers of women between 20 and 69 years in Finland in 2022 were breast, colon and rectum and melanoma of skin. The highest mortality for women was for breast, colon and rectum and lower respiratory system (lung, trachea) cancers [9]. In Finland, similar to other Nordic countries, early diagnosis and improved treatment have increased 5-year age-standardised relative cancer survival of women for 73% in 2017–2021 compared to 38% in 1972–1976 [11].

In Finland, fertility estimate statistics have been available for men only since the 1990s, so in the present study, we focus on the trends of the children with maternal cancer and not paternal cancers, also considering the overall significance of the maternal role in the childcare and development.

In this study, using population-based registry rates, we aim to estimate model-based trends by cancer type in the number and rate of prevalent and new children under 18 years of age for mothers diagnosed at ages 15 to 66 and the number and rate of prevalent and new maternal orphans by cancer type for mothers who died of cancer in 1968–2022 in Finland.

## Material and methods

The present observational study used national population-based registry rates to estimate indirect group-level model-based trends. Data for incident cancers between 1953 and 2022 were obtained from the Finnish Cancer Registry (FCR) [12, 13]. Cancer deaths (1953–2022) for women aged 15 to 66 years old, fertility rates (1951–2022) for women aged 15 to 49 years and mortality rates (1951–2022) for children aged 0 to 17 years were obtained from Statistics Finland [4]. The annual number of new children whose mother was diagnosed with cancer or died from cancer was estimated from 1968 to 2022 due to fertility rates being available from 1951 to 2022. The corresponding prevalent numbers were estimated from 1970 to 2022 because cancer cases and cancer deaths were available from 1953 to 2022.

For estimation of the annual number of new orphans, prevalent number of orphans, the number of new children whose mother was diagnosed with cancer and prevalent number of children (aged 0–17 years) with mother ever diagnosed with cancer, we used an alternated version of the methodology introduced in Guida et al. [8] (Supplementary File 1 Statistical methods). The relative risk for parity refers to the cancer risk of women who have given birth reference group being women who have not given birth [8] (Supplementary File 1). The relative risks (RRs) are assumed to be constant with respect to maternal age. The estimated model-based absolute numbers of children affected by maternal cancer are relevant, alongside age-standardised estimates, for assessing the burden on the healthcare system from a public health perspective.

We reported the average number of new and prevalent children with maternal cancer and orphans due to maternal cancer and the respective average age-standardised rates per 100,000 children (using World Standard Population 2000 in 1-year age groups) for 5-year periods at the start and end of the study period for all cancers combined, in addition to 5-year periods around estimated changepoints (Table 1). We estimated changepoints and piecewise trends (annual percentage change, annual percent changes (APC) and their 95% confidence intervals (CI) with programming language R (version 4.3.2) using Poisson regression (R-segmented version 2.1.1). The summary statistics are also reported separately for the year 2022. The average number of new and prevalent children with maternal cancer and the average number of new and prevalent orphans are reported by cancer type for 5-year intervals at the start (1968–1972), middle (1993–1997) and end (2018–2022) of the study period in Table 2. The first interval is limited to 1970–1972 for a prevalent number of children with maternal cancer and a prevalent number of orphans due to lack of data on incident cancers and cancer deaths from 1951 to 1952.

**Table 1:** Estimated number of new (1968–2022) and prevalent (1970–2022) children whose mother was diagnosed with cancer or who were maternal orphans due to cancer and the corresponding age-standardised rates (World 2000, per 100.000), in 2022 and 5-year averages at the start and end of the study period and around estimated changepoints.

Population quantity		First period	Average value^2^	Change point^1^	Average value^2^	Change point^2^	Average value^2^	Last period	Average value^2^	Value in 2022
New children with maternal cancer	Number of children	1968–1972	1,631	1984	1,332	1996	1,874	2018–2022	2,309	2,334
	Age-standardised rate	1968–1972	112.5	1984	112.7	1996	159.5	2018–2022	215.0	218.4
Prevalent children with maternal cancer	Number of children	1970–1974	11,453	1986	8,752	1998	12,670	2018–2022	16,396	16,803
	Age-standardised prevalence	1970–1974	796.9	1985	743.6	1999	1095.7	2018–2022	1494.0	1522.4
New orphans^1^	Number of orphans	1968–1972	655	1985	342	1997	369	2018–2022	264	285
	Age-standardised rate	1968–1972	44.9	1985	29.2	1998	31.7	2018–2022	24.4	26.4
Prevalent orphans^1^	Number of orphans	1970–1974	4,625	1987	2,281	1999	2,535	2018–2022	1,875	1,850
	Age-standardised prevalence	1970–1974	320.6	1986	201.2	2001	214.0	2018–2022	170.5	166.7

1 C00–96, D09.0–1, D32–33, D41–43, D45–47, D76, excluding C44

2 5-year average in the first and last period and around changepoints.

**Table 2:** Estimated average annual number of new children (1968–2022) and prevalent children (1970–2022) whose mother was diagnosed with cancer or who were maternal orphans due to cancer, presented by cancer type.

Cancer	Period	Average annual number of new children whose mother was diagnosed with cancer	Average number of children with mother diagnosed ever with cancer^1^	Average annual number of new orphans	Average prevalent number of orphans^1^
Brain, meninges and CNS (C70–72, D32–33, D42–43)	2018–2022	156	1,206	22	202
	1993–1997	131	935	30	265
	1968–1972	81	662	36	358
Breast (C50)	2018–2022	917	5,948	83	546
	1993–1997	762	4,393	125	738
	1968–1972	481	3,128	148	960
Colon and rectum (C18–20)	2018–2022	123	826	22	129
	1993–1997	92	554	20	127
	1968–1972	89	554	44	249
Female genital organs (C51–58)	2018–2022	294	2,100	35	272
	1993–1997	242	1,449	37	246
	1968–1972	440	3,482	124	986
Gallbladder, bile ducts (C23–24)	2018–2022	5	36	3	22
	1993–1997	8	39	4	26
	1968–1972	9	49	8	41
Haematological (C81–96, D45–47, D76)	2018–2022	173	1,329	15	130
	1993–1997	137	1,021	34	310
	1968–1972	119	1,003	79	699
Kidney (C64)	2018–2022	31	186	2	14
	1993–1997	27	166	6	36
	1968–1972	29	173	14	81
Liver (C22)	2018–2022	9	56	8	38
	1993–1997	5	44	6	34
	1968–1972	8	46	8	42
Lung, trachea (C33–34)	2018–2022	39	213	16	98
	1993–1997	36	198	22	121
	1968–1972	32	185	22	130
Melanoma of the skin (C43)	2018–2022	219	1,835	7	57
	1993–1997	93	738	10	78
	1968–1972	55	396	17	126
Pancreas (C25)	2018–2022	27	158	14	75
	1993–1997	15	93	13	73
	1968–1972	20	112	18	101
Stomach (C16)	2018–2022	23	163	10	84
	1993–1997	35	280	19	173
	1968–1972	89	674	72	562
Thyroid gland (C73)	2018–2022	157	1,441	2	8
	1993–1997	140	1,015	1	11
	1968–1972	52	326	5	38
Other (C00–15, C17, C21, C26, C30–32, C37–41, C45–49, C65–69, C74–80, D09.0–1, D41.1–9)	2018–2022	134	900	26	200
	1993–1997	104	693	33	243
	1968–1972	127	964	61	473

1 The first interval is 1970–1972.

**Figure 1:**
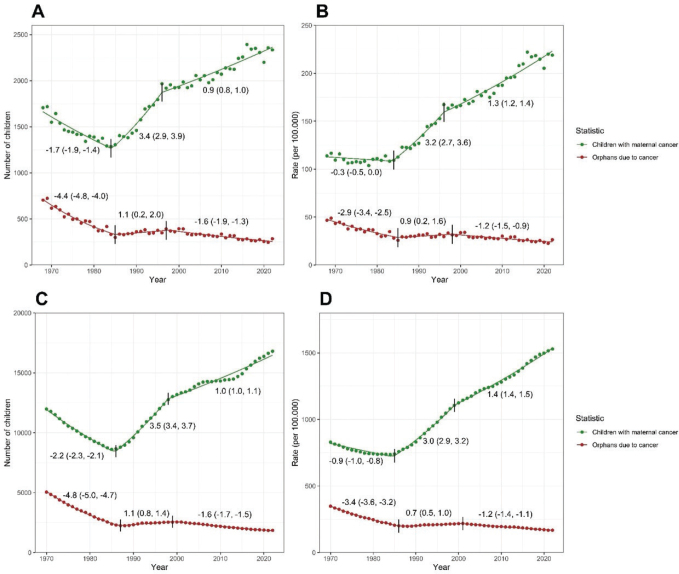
Estimated piecewise annual percentage changes (APCs) in the number of new children (A), age-standardised rate of new children (B), prevalent number of children (C) and age-standardised prevalence rate of children under 18 years (D) whose mother was diagnosed with cancer (children with maternal cancer) and orphans due to maternal cancer in Finland (between 1968 and 2022 in annual new numbers; 1970–2022 in prevalent numbers).

Changepoints and piecewise trends were estimated for the number of new children with maternal cancer and the number of new orphans (Figure 1A), the rate of new children with maternal cancer and the rate of new orphans (Figure 1B), the prevalent number of children with maternal cancer and the prevalent number of orphans (Figure 1C) and the prevalence rate of children with maternal cancer and the prevalence rate of orphans (Figure 1D), for selected cancer types and all cancers combined (Appendix Tables 1 and 2). Estimations were conducted for rates both adjusted and unadjusted for mothers’ age (age groups: 15–24, 25–34, 35–44, 45–54 and 55–66 years) to take into account the changes in the age distribution of mothers of children under 18 years over time. The yearly age distribution of mothers with children under 18 years in Finland in 1970–2022 is presented in 5-year age groups in Appendix Figure 1. All rates were adjusted for the child’s age (6-year age groups: 0–5, 6–11 and 12–17 years). The number of changepoints for each time series was selected using Bayesian Information Criterion (BIC) [14] constrained to a maximum of two changepoints. APCs are reported separately for each section defined by the optimised changepoints in Appendix Tables 1 and 2 to describe the piecewise trends.

The selected cancer types and their ICD-10 codes are presented in Table 2. The ‘Other’ cancers category was created using a cut-off limit of cancers approximately less than 1% incidence and 1% in mortality cases.

**Figure 2:**
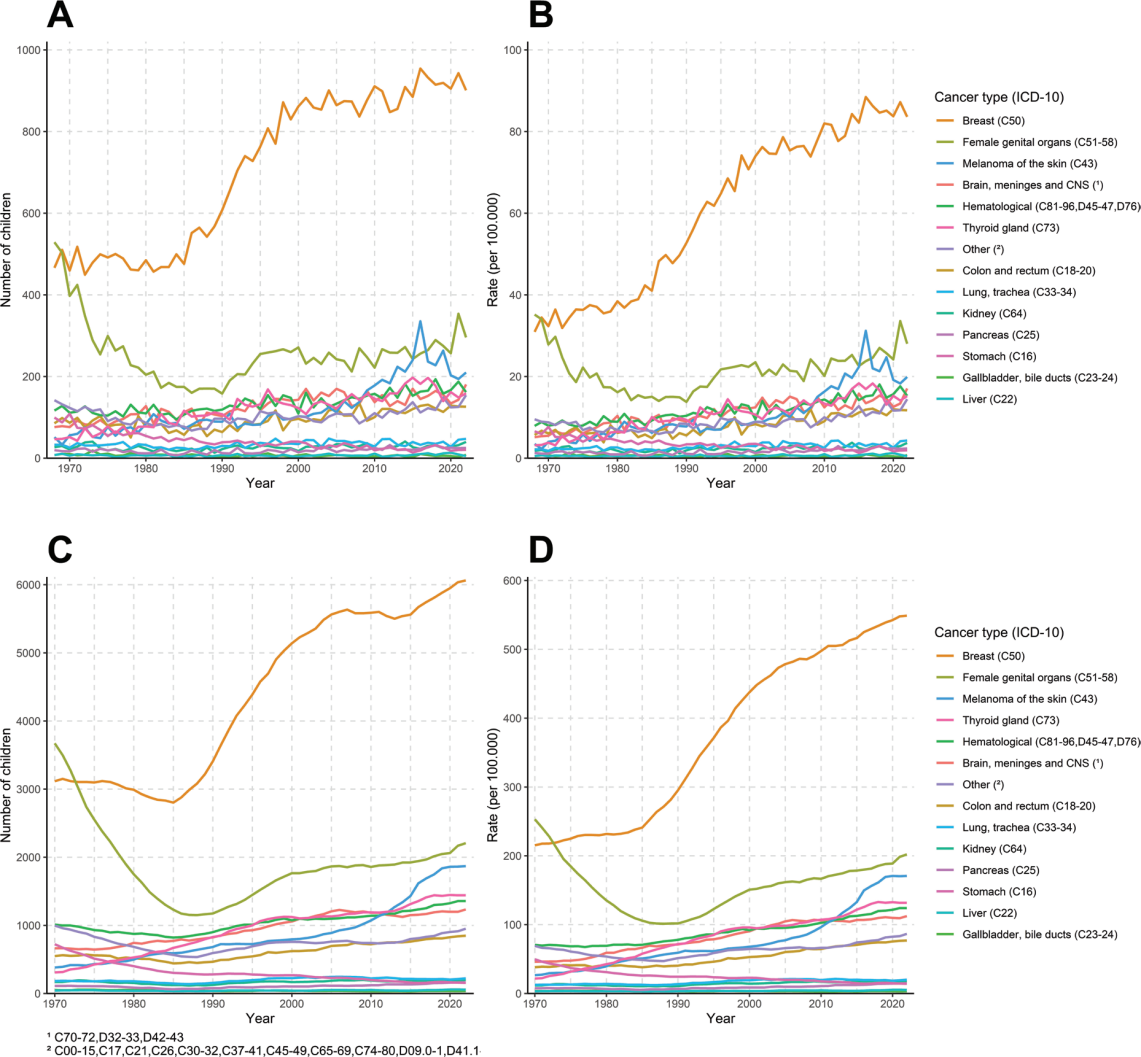
Estimated annual number (A) and age-standardised rate of new children (B) (1968–2022), prevalent number (C) and age-standardised prevalence rate (D) (1970–2022) of children whose mother was diagnosed with cancer presented by cancer type.

**Figure 3:**
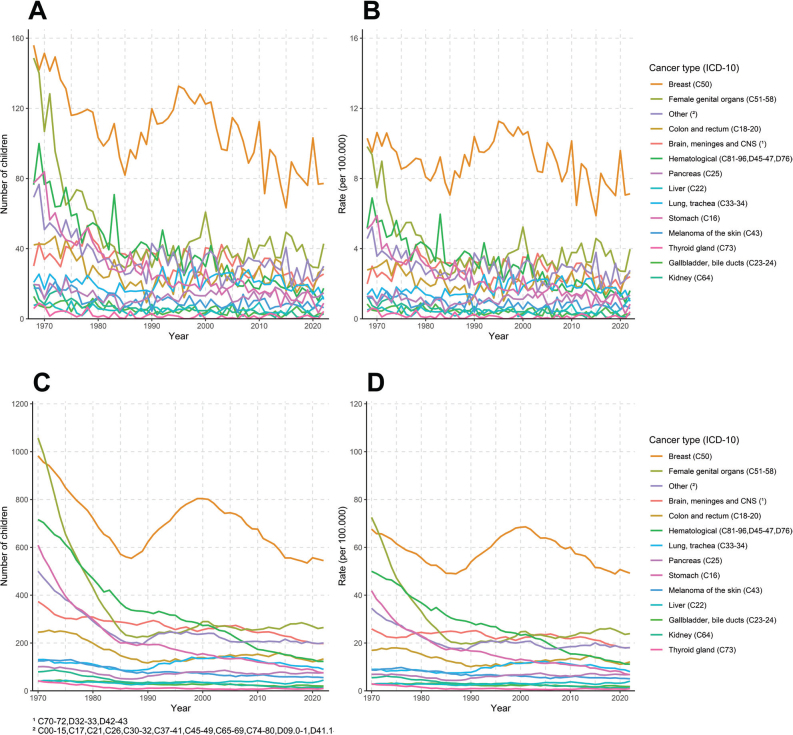
Estimated annual number (A) and age-standardised rate of new orphans (B) (1968–2022), prevalent number (C) and age-standardised prevalence rate (D) (1970–2022) of orphans due to maternal cancer presented by cancer type.

We presented trends of estimated annual number (Figure 2A) and age-standardised rate of new children (Figure 2B) (1968–2022) and prevalent number (Figure 2C) and age-standardised prevalence rate of children under 18 years (Figure 2D) (1970–2022) whose mother was diagnosed with selected cancer types. Similarly, we estimated the annual number (Figure 3A) and age-standardised rate of new orphans (Figure 3B) (1968–2022) and the prevalent number (Figure 3C) and age-standardised prevalence rate of orphans under 18 years (Figure 3D) (1970–2022) due to maternal cancer mortality presented by cancer type.

## Results

### Children with maternal cancers

The estimated number of **new children, whose mother was diagnosed with cancer** in 2022, was 2,334, and the age-standardised rate was 218 per 100,000 children. In the period 1994–1998 around the latest changepoint, the average annual number of new children was 1,874 (age-standardised rate 160/100,000) (Table 1). The annual percentage change in the number of new children whose mother was diagnosed with cancer was 0.9% since 1996. In the five-year period around the latest changepoint (1994–1998), the average annual number of new children was 1,874 (age-standardised rate 160/100,000). The trend of the number of new children whose mother was diagnosed with cancer was not linear over time (Figure 1A). The age-adjusted (maternal age) annual percent change rate of new children whose mother was diagnosed with cancer was 1.3% in 2007–2022 (Table 3).

**Table 3:** Changepoint section-wise annual percent changes (APC) and their 95% confidence intervals (CI) of number of new children and rate of new children (unadjusted and adjusted for mother’s age) whose mother was diagnosed with cancer by cancer type in 1968–2022.

Cancer	Number of new children (1968–2022)		Rate of new children, unadjusted for mother’s age (1968–2022)		Rate of new children, adjusted for mother’s age (1970–2022)	
	Changepoint section	APC (95% CI)	Changepoint section	APC (95% CI)	Changepoint section	APC (95% CI)
All sites together (C00–43, C45–59, C64–96, D09.0–1, D32–33, D41–43, D45–47, D76)	1996–2022	0.9 (0.8, 1.0)	1996–2022	1.3 (1.2, 1.4)	2007–2022	1.3 (1.0, 1.5)
	1984–1996	3.4 (2.9, 3.9)	1984–1996	3.2 (2.7, 3.6)	1997–2007	0.4 (–0.2, 1.0)
	1968–1984	–1.7 (–1.9, –1.4)	1968–1984	–0.3 (–0.5, 0.0)	1970–1997	1.7 (1.5, 1.8)
Brain, meninges and CNS (C70–72, D32–33, D42–43)	2001–2022	0.1 (–0.4, 0.7)	2001–2022	0.4 (–0.1, 0.9)	2000–2022	0.3 (–0.2, 0.8)
	1968–2001	1.9 (1.6, 2.3)	1968–2001	2.5 (2.1, 2.8)	1970–2000	2.3 (1.9, 2.7)
Breast (C50)	1998–2022	0.3 (0.2, 0.5)	1998–2022	0.7 (0.6, 0.9)	1995–2022	0.3 (0.2, 0.5)
	1983–1998	4.1 (3.6, 4.6)	1982–1998	4.0 (3.6, 4.4)	1970–1995	3.0 (2.8, 3.3)
	1968–1983	–0.3 (–0.8, 0.2)	1968–1982	1.2 (0.6, 1.8)		
Colon and rectum (C18–20)	1985–2022	1.8 (1.5, 2.1)	1985–2022	2.0 (1.7, 2.3)	1970–2022	1.1 (0.9, 1.3)
	1968–1985	–1.7 (–2.8, –0.7)	1968–1985	–0.4 (–1.4, 0.5)		
Female genital organs (C51–58)	1996–2022	0.6 (0.3, 0.9)	1996–2022	1.0 (0.7, 1.3)	1982–2022	0.9 (0.8, 1.1)
	1985–1996	4.4 (3.0, 5.8)	1984–1996	3.6 (2.5, 4.7)	1970–1982	–3.9 (–4.8, –3.1)
	1968–1985	–6.9 (–7.4, –6.3)	1968–1984	–5.7 (–6.3, –5.1)		
Gallbladder, bile ducts (C23–24)	1968–2022	–1.4 (–2.0, –0.7)	1968–2022	–0.8 (–1.4, –0.2)	1970–2022	–1.3 (–2.0, –0.7)
Haematological (C81–96, D45–47, D76)	1983–2022	1.2 (0.9, 1.4)	1968–2022	1.4 (1.2, 1.5)	1970–2022	1.2 (1.1, 1.4)
	1968–1983	–0.3 (–1.2, 0.7)				
Kidney (C64)	1981–2022	1.0 (0.5, 1.5)	1968–2022	0.9 (0.6, 1.2)	1970–2022	0.5 (0.2, 0.8)
	1968–1981	–2.7 (–5.5, 0.1)				
Liver (C22)	1968–2022	0.5 (–0.1, 1.1)	1968–2022	1.0 (0.3, 1.6)	1970–2022	0.9 (0.2, 1.6)
Lung, trachea (C33–34)	1997–2022	–0.2 (–1.0, 0.6)	1968–2022	1.1 (0.9, 1.4)	1997–2022	–0.6 (–1.5, 0.3)
	1987–1997	4.8 (0.8, 9.0)				
					1970–1997	1.7 (0.9, 2.6)
	1968–1987	–1.9 (–3.3, –0.4)				
Melanoma of the skin (C43)	2016–2022	–5.8 (–8.7, –2.7)	2016–2022	–5.3 (–8.3, –2.3)	2016–2022	–5.9 (–8.8, –2.9)
	2006–2016	8.4 (7.0, 9.9)	2005–2016	8.5 (7.0, 10.0)	2007–2016	9.7 (8.0, 11.4)
	1968–2006	2.0 (1.6, 2.3)	1968–2005	2.5 (2.1, 2.8)	1970–2007	2.2 (1.9, 2.5)
Pancreas (C25)	1984–2022	2.0 (1.4, 2.7)	1984–2022	2.3 (1.6, 2.9)	1970–2022	0.9 (0.5, 1.3)
	1968–1984	–3.3 (–5.7, –0.8)	1968–1984	–1.7 (–4.1, 0.6)		
Stomach (C16)	1981–2022	–2.1 (–2.6, –1.7)	1968–2022	–2.3 (–2.5, –2.0)	1970–2022	–2.6 (–2.8, –2.3)
	1968–1981	–5.2 (–6.7, –3.7)				
Thyroid gland (C73)	2017–2022	–3.4 (–8.0, 1.5)	2017–2022	–2.8 (–7.5, 2.1)	1985–2022	1.5 (1.3, 1.8)
	1987–2017	1.6 (1.3, 2.0)	1985–2017	1.9 (1.6, 2.3)	1970–1985	6.3 (4.9, 7.6)
	1968–1987	4.6 (3.6, 5.6)	1968–1985	6.0 (4.9, 7.2)		

CNS, central nervous system.

Over time since 1970, the number of children and number of young mothers has decreased, which has increased the proportion of older mothers with children under 18 years (Appendix Figure 1). The estimated annual percentage change for the rate of new children whose mother was diagnosed with cancer increased since 1984 (Figure 1B) and the prevalent adjusted annual change (1970–2022) in rate was 1.3% (Table 3).

Maternal diagnosis of breast cancer (*n* = 917, 40%) was the major reason for the average number of new children whose mother was diagnosed with cancer in 2018–2022; the second cancer type, with significantly fewer cases, was female genital organ cancers (*n* = 294, 12.7%) and the third was skin melanoma (*n* = 219, 9.5%) (Table 2). The estimated number and rate of new children whose mother was diagnosed with female genital organ cancers were significantly decreased over time, whereas the estimated number of children whose mother was diagnosed with breast cancer was substantially increased (Figure 2A and B). During the latest changepoint sections, the unadjusted rate of new children whose mother was diagnosed increased annually for pancreatic cancer 2.3% in 1984–2022, for colon and rectal cancers 2% in 1985–2022, for haematological cancers 1.4% in 1968–2022, for female genital organ cancers 1.0% in 1996–2022 and for breast cancer 0.7% in 1998–2022, whereas there was 2.3% decrease for stomach cancer in 1968–2022 (Table 3). The unadjusted rate of new children whose mother was diagnosed with skin melanoma increased annually by 8.5% in 2005–2016, followed by a 5.3% decrease in 2016–2022 (Table 3).

The estimated number of **children (prevalent), whose mother has ever been diagnosed with cancer** in 2022, was 16,803, and the age-standardised rate was 1,522 per 100,000 children (Table 1). The number of prevalent children whose mother has ever been diagnosed with cancer increased annually by 1.0% since 1998 (Figure 1C).

The estimated rate of children whose mother has ever been diagnosed with cancer has increased 1.4% annually since 1999 (Figure 1D).

Maternal diagnosis of breast cancer (*n* = 5,948, 36.3%) was also the major reason for the estimated **average number of prevalent children whose mother was ever diagnosed with cancer** in 2018–2022; the second most common cancer type, with significantly less cases, was female genital organ cancers (*n* = 2,100, 12.8%) and the third was skin melanoma (*n* = 1,835, 11%) (Table 2). Prevalent annual numbers and rate of children whose mother was diagnosed with cancer have increased due to a significant increase in the diagnosis of breast cancer compared to the 1968–1972 period, when breast cancer was the second cause after female genital organ cancers (Figure 2C and D).

### Orphans due to maternal cancer death

In 2022, we estimated 285 **new maternal orphans.** The age-standardised rate was 26 per 100,000 orphan children due to maternal cancer death (Table 1). The number of new maternal orphans has decreased 1.6% annually since 1997 (Figure 1A). The unadjusted rate of new orphans had a 1.2% annual decrease since 1998 (Figure 1B, Table 4).

**Table 4:** Changepoint section-wise annual percent changes (APC) and their 95% confidence intervals of number of new maternal orphans and rate of new maternal orphans (unadjusted and adjusted for mother’s age) due to cancer by cancer type.

Cancer	Number of new orphans (1968–2022)		Rate of new orphans, unadjusted for mother’s age (1968–2022)		Rate of new orphans, adjusted for mother’s age (1970–2022)	
	Change point section	APC (95% CI)	Change point section	APC (95% CI)	Change point section	APC (95% CI)
All sites together (C00–43, C45–59, C64–96, D09.0–1, D32–33, D41–43, D45–47, D76)	1997–2022	–1.6 (–1.9, –1.3)	1998–2022	–1.2 (–1.5, –0.9)	1998–2022	–1.7 (–2.0, –1.4)
	1985–1997	1.1 (0.2, 2.0)	1985–1998	0.9 (0.2, 1.6)	1970–1998	–1.0 (–1.2, –0.8)
	1968–1985	–4.4 (–4.8, –4.0)	1968–1985	–2.9 (–3.4, –2.5)		
Brain, meninges and CNS (C70–72, D32–33, D42–43)	1968–2022	–0.9 (–1.2, –0.6)	1978–2022	–0.9 (–1.3, –0.5)	1978–2022	–1.3 (–1.7, –0.9)
			1968–1978	3.9 (0.3, 7.6)	1970–1978	5.8 (0.8, 11.2)
Breast (C50)	1995–2022	–2.1 (–2.5, –1.6)	1995–2022	–1.7 (–2.2, –1.2)	1996–2022	–2.3 (–2.8, –1.7)
	1985–1995	3.4 (1.2, 5.5)	1985–1995	3.1 (1.2, 5.0)	1970–1996	0.4 (–0.1, 0.8)
	1968–1985	–3.1 (–3.9, –2.3)	1968–1985	–1.6 (–2.4, –0.7)		
Colon and rectum (C18–20)	1987–2022	0.6 (–0.1, 1.3)	1989–2022	0.9 (0.1, 1.7)	1970–2022	–1.0 (–1.3, –0.6)
	1968–1987	–4.4 (–5.7, –3.1)	1968–1989	–3.0 (–4.1, –1.8)		
Female genital organs (C51–58)	1984–2022	0.3 (–0.2, 0.7)	1984–2022	0.5 (0.0, 1.0)	1984–2022	–0.5 (–0.9, 0.0)
	1968–1984	–8.4 (–9.5, –7.2)	1968–1984	–7.0 (–8.2, –5.9)	1970–1984	–5.1 (–6.5, –3.7)
Gallbladder, bile ducts (C23–24)	1968–2022	–2.2 (–3.0, –1.5)	1968–2022	–1.6 (–2.4, –0.9)	1970–2022	–2.0 (–2.8, –1.2)
Haematological (C81–96, D45–47, D76)	1968–2022	–3.1 (–3.4, –2.8)	1968–2022	–2.5 (–2.8, –2.3)	1992–2022	–3.9 (–4.7, –3.1)
					1970–1992	–1.6 (–2.5, –0.7)
Kidney (C64)	1968–2022	–3.4 (–4.1, –2.7)	1968–2022	–3.0 (–3.7, –2.3)	1970–2022	–3.4 (–4.1, –2.6)
Liver (C22)	1986–2022	1.6 (0.3, 2.9)	1968–2022	0.7 (0.0, 1.4)	1970–2022	0.5 (–0.3, 1.2)
	1968–1986	–3.8 (–7.2, –0.2)				
Lung, trachea (C33–34)	2004–2022	–2.9 (–4.7, –1.1)	2008–2022	–2.8 (–5.7, 0.1)	1995–2022	–2.1 (–3.1, –1.0)
	1985–2004	2.6 (0.6, 4.6)				
	1968–1985	–1.9 (–3.9, 0.1)	1968–2008	1.2 (0.6, 1.8)	1970–1995	2.2 (1.1, 3.4)
Melanoma of the skin (C43)	1968–2022	–1.5 (–2.0, –1.0)	1968–2022	–1.1 (–1.6, –0.5)	1970–2022	–1.6 (–2.1, –1.0)
Pancreas (C25)	1984–2022	0.7 (–0.1, 1.5)	1968–2022	0.2 (–0.2, 0.7)	1970–2022	–0.2 (–0.7, 0.2)
	1968–1984	–3.4 (–6.0, –0.7)				
Stomach (C16)	1982–2022	–2.8 (–3.4, –2.2)	1968–2022	–3.1 (–3.4, –2.8)	1970–2022	–3.3 (–3.7, –3.0)
	1968–1982	–6.1 (–7.6, –4.5)				
Thyroid gland (C73)	1997–2022	2.4 (–2.6, 7.8)	1968–2022	–2.8 (–4.1, –1.5)	1970–2022	–2.7 (–4.1, –1.3)
	1968–1997	–6.9 (–9.7, –4.1)				

CNS, central nervous system.

Maternal deaths from breast cancer were the single largest cause of **new maternal orphans** (*n* = 83, 31.4%), followed by female genital organ cancers (*n* = 35, 13.2%), colon and rectum (*n* = 22, 8.3%) and brain, meninges and central nervous system (CNS) cancers (*n* = 22, 8.3%) in 2018–2022. Maternal orphans due to deaths from skin melanoma were minor (*n* = 7, 2.5%) (Table 2). In 1968–1972, estimated deaths from breast cancer were the single largest cause of new maternal orphans (*n* = 148, 22.6%), the second cause was female genital organ cancers (*n* = 124, 18.9%) and the third was haematological cancers (*n* = 79, 12.1%) (Table 2). The decrease in estimated number and rate of new orphans over time by maternal cancer mortality type was profound as in general in all these cancers: breast, female genital organ and haematological cancers (Figure 3A and B). The annual percent change showed a 1.7% decrease in the unadjusted rate of new maternal orphans due to breast cancer death in 1995–2022 and a 2.5% decrease due to haematological cancer deaths in 1968–2022. There was a 5.1% annual decrease in the adjusted rate of new maternal orphans due to female genital cancer deaths in 1970–1984, which was stabilised to a 0.5% annual decrease in 1984–2022. There was also a profound 3.1% annual decrease in the adjusted rate of new maternal orphans in stomach cancer deaths, 3.0% in kidney cancer deaths and 2.8% in thyroid cancer deaths in 1968–2022 (Table 4).

In 2022, we estimated 1,850 **prevalent maternal orphans** (age-standardised 167/100,000) (Table 1). The prevalence rate of maternal orphans has decreased 1.2% annually since 2001 (Figure 1D). The estimated **prevalent annual number and rate of orphans** by maternal cancer death showed breast cancer and female genital organ cancers being the largest causes in 1970–1972, with a major decreasing trend towards the later interval 2017–2021 (Figure 3C and D).

## Discussion and conclusion

We sought to estimate how many children in Finland are affected by maternal cancer or cancer death and if this has changed over the past decades. More specifically, we estimated the model-based trend in the number and rate of prevalent and new children whose mother was diagnosed with cancer and prevalent and new maternal orphans due to cancer mortality by cancer type in 1968–2022. The estimated rate of children whose mother was diagnosed with cancer increased 1.3% annually since 1996. On the contrary, the estimated rate of new orphans due to maternal cancer mortality decreased 1.2% annually since 1998. In Finland, the estimated number of new orphans due to maternal cancer mortality decreased almost to half during the past 50 years.

Based on the FCR statistics, cancer cases among young adults remain relatively rare, but there is a noticeable increase in specific types such as skin melanoma and thyroid gland cancer in 20- to 29-year-old women since the 2000s. Women aged 30–49 years show a significant rise in breast cancer cases, the second most common cancer being skin melanoma and the third thyroid gland cancer [12]. In our study, the estimated number of new children whose mother was diagnosed with female genital organ cancers was significantly decreased over time, whereas the estimated number of new children whose mother was diagnosed with breast cancer was substantially increased. The age-adjusted increase in the rate of new children whose mother was diagnosed with thyroid gland cancer was 1.5% since 1985. Breast, female genital organ and skin melanoma cancers were estimated for being most frequently diagnosed prevalent and new cancer types for mothers who had children under the age of 18 years in 2018–2022. However, skin melanoma was less significant when the number of new orphans was estimated by maternal cancer mortality type. Maternal breast cancer was the main cancer for the estimated number of prevalent and new orphans, followed far behind by other cancer types such as female genital organ cancers. The estimated number of new and prevalent orphans due to maternal cancer death decreased since the 1990s for all maternal cancer types.

In our study in 2022, the rates of new and prevalent children with maternal cancer were 218 and 1,522 per 100,000, corresponding to 2,334 and 16,803 children. In 2022, the age-standardised rates of new and prevalent orphans were 26 and 167 per 100,000 children, corresponding to 285 and 1,850 orphans due to maternal cancer. The estimated rate of new orphans was comparable to earlier estimates for the year 2000 in the Northern Europe region (33 per 100,000 children) and, as expected, lower than the world estimate average (40 per 100,000 children) [8].

Cancer incidence has increased in women by 0.8% on average per year between 1992 and 2019 in Finland. Approximately 17,600 women were diagnosed with cancer in 2022 [9]. About five out of every hundred women are diagnosed with cancer before the age of 50 years and less than one out of every hundred women before the age of 30 years. The cancer burden increases markedly after age 50. Death due to breast cancer is the most common cancer death among women aged between 20 and 69 years (mortality 17.6/100,000 and 301 deaths in 2022) [12].

Early diagnosis and improved treatment increased 5-year age-standardised relative cancer survival of women in the 2020s compared to the 1970s in Finland, as in all Nordic Countries [11]. At the same time, demographic transitions occurred; the average age at first confinement in women in the high-income (Nordic) countries is increasing and fertility decreasing. According to Statistics Finland’s data on population changes, the average age of first-time biological mothers was 31.6 years in 2022, whereas it was 26.8 years in 1990 (5). Therefore, mothers of minor children appear older and more susceptible to cancer incidence, which increases with age.

Despite the increase in cancer cases among young adults, mortality rates have not risen. Advances in treatment have improved survival rates, but the treatments can be arduous and have both short-term and long-term impacts on quality of life. Cancer and cancer treatment affect working ability and income, physical functioning and sexuality and social relationships, for example marital relationship. Stressors in the family due to parental cancer may also affect children, who may not receive as much care and support due to diminished parental resources. For example, children may be obliged to be caretakers themselves in these circumstances.

The estimates of this study are pertinent because parental illness and death have been shown to have long-term impacts on child’s well-being, also in high HDI countries, including Northern Europe [5–7]. Parental cancer and cancer death affect children beyond just economical resources, also resulting in disadvantages in social and emotional well-being and general health. In three high-income Nordic countries (Sweden, Denmark and Finland), parental death of any natural causes during childhood and adolescence was found to be associated with an increased risk of natural and unnatural mortality that persisted into early adulthood [7]. Parental cancer and cancer death have been found to be associated with child’s lower educational attainment and lower income in early adulthood [5, 6]. A significant increase in the estimated number of children whose mother was diagnosed with cancer may impact socioeconomic health inequalities despite favourable decreasing trend for the estimated number of orphans caused by maternal cancer.

In Finland, population-based breast cancer screening started in 1987 for women aged 50–69 years [15] and cervical cancer screening in early 1970s in women aged between 30 and 65 years [10]. Breast cancer screening has been effective to reduce breast cancer mortality, but it is not covering fertility-aged women. Screening for cervical cancer has decreased cervical cancer-specific mortality also in younger women. National human papillomavirus (HPV) vaccination programme started in Finland in 2013 for girls aged 11–12 years and similarly for boys in 2020. Studies and mathematical models suggest that high vaccine coverage among young women can decrease HPV type-specific cervical cancer incidence by up to 91% [16, 17]. With fewer cases of cervical cancer, the mortality rate is also expected to decline. HPV vaccination programme in Finland is a crucial step towards the near elimination of cervical cancer in the future. Also new recommendations and considerations for expanding the breast cancer screening target population to younger women aged 45–49 years may benefit mothers with young children in the future [18].

The future burden of cancer incidence is expected to rise in most countries over the coming decades [2]. In Finland, the age-standardised cancer incidence from 2022 to 2040 is estimated to increase for women by 0.2% yearly. Women’s cancer deaths are estimated to decrease 0.7% on average during the same time. The rise in cancer cases is mainly due to the aging population, and the amount is estimated to grow by 63% in people over 75 years old [9]. This will create more demand for health care services overall and possibly affect the limited resources available. Diagnostic delays and access to adequate treatments at the right time are a concern in this situation, but the need for supportive services should also be taken into consideration.

Our data have some limitations. Registries were not employed to trace actual familial relationships but served as a basis for deriving population rates employed in indirect, model-based estimation. The key advantage of this approach is the availability of pre-existing data from a long period (fertility and mortality rates from 1951 and cancer incidence and cancer mortality rates from 1953) that can be readily utilised. Population registry data in Finland has deficiencies related to individual-level familial information during the first decades of the registry, with complete registry data on parents of children available only since 1975. Therefore, our initial objective has been to examine overall long-term average time series trends from a methodological perspective. In subsequent stages, the aim is to enrich the dataset and refine the understanding of the association and interrelations of various factors using individual-level data. We did not have data on the fertility history of women with cancer, and the analyses were conducted using population data on fertility rates in Finland. We followed the methodology of Guida et al. [8] to incorporate the associations between reproduction and cancer risks for breast, cervical and ovarian cancers, but otherwise assumed that the past fertility of women diagnosed with or who died from cancer did not differ from that of their birth cohort. The model-based estimates are not very robust, as the RRs directly affect the estimated numbers, that is excluding the RRs (relative risk for parity to the cancer risk of women who have given birth) from the model would increase the current estimated total number of children affected by maternal ovarian cancer (RR = 0.8) for the whole period 1968–2022 by 25%, from 4,043 to 5,054, and the respective rate per 100.000 children from 6.3 to 7.9. Breast, cervical and ovarian cancers were the only ones whose risk has been established as significantly associated with parity. We restricted data to mothers excluding fathers, for which the fertility data were not available until the 1990s. Orphans due to cancer deaths in men would require different methodology and would also differ from women due to men being reproductive to a significantly higher age. However, the strength of the methodology is that for the estimation we could use long-term good-quality population register data, which has been collected by Statistics Finland over decades. Fertility and mortality rates are based on the production of Official Statistics of Finland (OSF), which must be continuous and of high quality and must commit to quality criteria, and we have excellent coverage of cancer deaths.

In conclusion, the estimated decreasing trend in cancer mortality among mothers with children under 18 years of age has benefited children and resulted in less orphans over the years. However, the increase in cancer incidence among mothers with minor children indicates the opposite trend, and more children have become affected by maternal cancer burden and its possible psychosocial consequences. This should be considered closely when applying strategies, aligned with the EU Mission for Cancer, for those affected by cancer, including their families, to live longer and better lives.

## Supplementary Material



## Data Availability

Statistics Finland Data is openly accessed for fertility rate and mortality rate on the StatFin database. Finnish Cancer Registry data for incidence and mortality are accessed via research permission by the Social and Health Data Permit Authority (Findata) in Finland.
