# Supplementary material for: Model-based trends in the estimated number of children affected by maternal cancer diagnosis or death in Finland in 1968–2022
Source: Acta Oncol. 2025 Sep 23;64:44072. doi: 10.2340/1651-226X.2025.44072 (PMC12476057; doi:10.2340/1651-226X.2025.44072)
Supplement: Supplementary file 1 [file AO-64-44072-s1.pdf]

Supplementary material has been published as submitted. It has not been copyedited, or typeset by Acta Oncologica

Appendix Figure 1. Number of children in Finland by mother's age group (1970–2022).

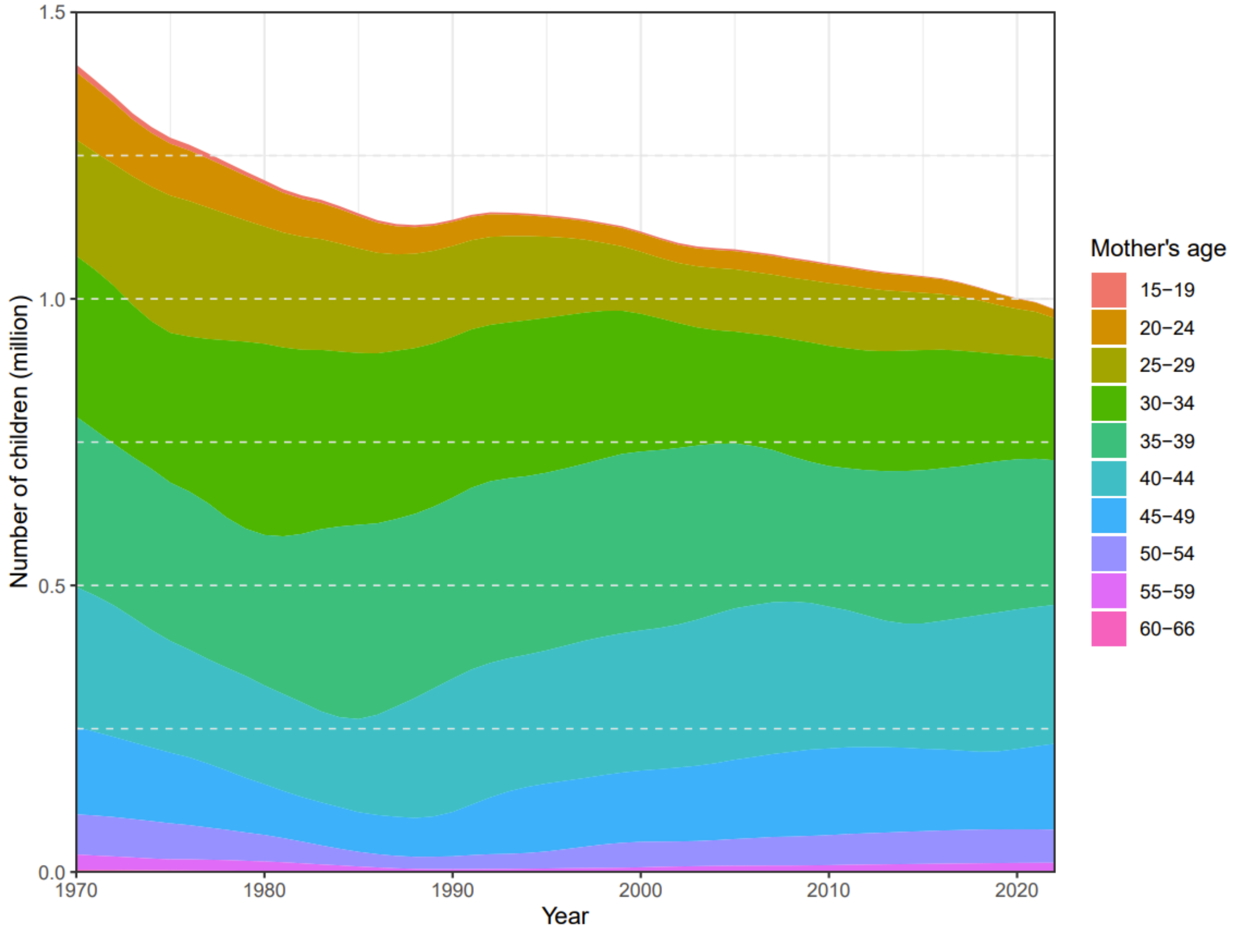

Supplement 1– Statistical methods

For the estimation of the number of orphans we used an altered version of the methodology introduced by Guida et al. [8] and further applied it to calculate the number of children whose mother was diagnosed with cancer and the age-standardized rates for these estimates. The estimates for new cases were calculated for each year from 1968 to 2022 and prevalent cases from 1970 to 2022.

The data included fertility rates $FR_{y,ma}$ by calendar year y (1951, …, 2022) and mother's age ma (15, …, 49 years), mortality rates $M_{y,ca}$ by calendar year y (1951, …, 2022) and child's age ca (0, …, 17 years), and cancer deaths $D_{y,ma,t}$, by mother's age ma (15, …, 66) and cancer type t for calendar years y (1953, …, 2022) from Statistics Finland, numbers of observed cancer

cases $C_{y,ma,t}$, by mother's age *ma* (15, …, 66) and cancer type *t* for calendar years y (1953, …, 2022) from the Finnish Cancer Registry, and the relative risks of parity on specific cancers

$$RR_{t,ma} = \begin{cases} 1.1, & \text{for cervix uteri} \\ 0.8, & \text{for ovary} \\ 0.93, & \text{for breast and mother's age over 50 years} \\ 1, & \text{otherwise.} \end{cases}$$

The mortality rates used were the year and age-wise averages of the mortality rates of boys and girls.

Probability that a child born in year yob is alive in year y, when their mother has not died from cancer is denoted by $P(alive_y|yob)$ and was estimated on the basis of the mortality rates by $exp\{-\sum_{i=yob}^{y-1} M_{i,i-yob}\}$.

Probability that a child born in year yob is alive in year y, given that their mother died of cancer in year md is denoted by $P(alive_y|yob, md)$ and was estimated by $= exp\{-\sum_{i=yob}^{md-1} M_{i,i-yob} - 1.25 \cdot \sum_{i=md}^{y-1} M_{i,i-yob}\}$. The coefficient 1.25 is the mortality HR for children who have lost a parent, based on data from three Nordic countries (methodology presented in Li et al. [7], and Guida et. al. 2022) [8].

For cases of new orphans and new children with maternal cancer we estimated the **A**verage number of living **C**hildren by mother's age *ma* (15, …, 66 years) and calendar year *y* (1968, …, 2022) given that the mother has been diagnosed with or died of cancer of type t:

$$AC_{y,ma,t} = \sum_{i=y-17}^{y} RR_{t,ma} \cdot FR_{i,ma-(y-i)} \cdot P(alive_y|i).$$

In the case of total orphans, we instead calculated the **A**verage number of living **O**rphans (**P**revalent) by calendar year *y* (1970, …, 2022) as

$$AOP_{y,ma,t} = \sum_{md=y-17}^{y} \sum_{i=y-17}^{md} RR_{t,ma-(y-md)} \cdot FR_{i,ma-(y-i)} \cdot P(alive_y|i, md)$$

and in the case of total children with mother diagnosed ever during child's lifetime we estimate the **A**verage number of **C**hildren (**P**revalent) by calendar year *y* (1970, …, 2022) as

$$ACP_{y,ma,t} = \sum_{md=y-17}^{y} \sum_{i=y-17}^{md} RR_{t,ma-(y-md)} \cdot FR_{i,ma-(y-i)} \cdot P(alive_y|i).$$

Number of **N**ew children whose mother was **D**iagnosed with cancer type t by year y (1968, …, 2022):

$$ND_{y,t} = \sum_{ma=15}^{66} C_{y,ma,t} \cdot AC_{y,ma,t}.$$

Number of **N**ew **O**rphans due to cancer type t by year y (1968, …, 2022):

$$NO_{y,t} = \sum_{ma=15}^{66} D_{y,ma,t} \cdot AC_{y,ma,t}.$$

Number of **T**otal children whose mother has been **D**iagnosed with cancer type t during child's lifetime by year y (1970, …, 2022):

$$TD_{y,t} = \sum_{ma=15}^{66} C_{y,ma,t} \cdot ACP_{y,ma,t}.$$

Number of **T**otal **O**rphans due to cancer type t by year y (1970, …, 2022):

$$TO_{y,t} = \sum_{ma=15}^{66} D_{y,ma,t} \cdot AOP_{y,ma,t}.$$

The age-standardized rates for maternal orphanhood due to cancer,

$$ASR_y = 100.000 \cdot \sum_{a=0}^{17} w_a \cdot \frac{NOA_{a,y}}{pop_{a,y}},$$

where w_a is the weight for the 1-year age group a, as calculated from the 2000 world population, $pop_{a,y}$ is the mean population of age group a in year y, and $NOA_{a,y}$ is the age-specific number of new orphans due to cancer in year y:

$$NOA_{a,y} = \sum_{t} \sum_{ma=15+a}^{66-(17-a)} C_{y,ma,t} \cdot RR_{t,ma} \cdot FR_{y-a,ma-a} \cdot P\left(alive_y \middle| y-a\right).$$
